# Rate of return to work in patients with stroke under the health and employment support program of Rosai hospitals in Japan

**DOI:** 10.1038/s41598-023-43162-2

**Published:** 2023-09-22

**Authors:** Takeru Umemura, Kenji Hachisuka, Satoru Saeki, Shigeru Nishizawa, Junkoh Yamamoto

**Affiliations:** 1 Department of Neurosurgery, University of Occupational and Environmental Health, Kitakyushu, Japan; 2 Department of Neurosurgery, Moji Medical Center, Kyushu Rosai Hospital, Kitakyushu, Japan; 3 Department of Rehabilitation Medicine, Moji Medical Center, Kyushu Rosai Hospital, Kitakyushu, Japan; 4 Department of Rehabilitation Medicine, University of Occupational and Environmental Health, Kitakyushu, Japan

**Keywords:** Neuroscience, Diseases, Medical research, Neurology

## Abstract

To facilitate return to work (RTW) in patients with stroke, a health and employment support (HES) program was started at Rosai hospitals in Japan. This study aimed to determine the rate of RTW in patients with stroke under this support program. We collected demographic and clinical data of patients with stroke from the implementation reports of the HES program. The program provided coordinated dual support, such as acute medical treatments, and stroke and vocational rehabilitation on the medical side, and management and support on the workplace side. The primary endpoint was RTW. Successful and unsuccessful RTW were examined using the χ^2^ test. The RTW rate curves were analyzed using the Kaplan–Meier method. We enrolled 483 patients; 355 (73%) and 128 (27%) patients had successful and unsuccessful RTW, respectively. Stroke types, neurological findings, and activities of daily living were significant factors for RTW. The Kaplan–Meier method revealed that left hemiplegia, right hemiplegia, and neuropsychological deficits, except for combined disability (hemiplegia with neuropsychological deficits), had similar RTW curves with an RTW rate of > 70%.

## Introduction

The ability to return to work (RTW) is the primary goal of treatment and rehabilitation in patients with stroke, with post-stroke RTW rates of 19–73%.^1^ Treger et al.^1^ reported that factors positively related to RTW were age less than 65 years, higher education level, and white-collar employment; the negative predictor was the severity of stroke. Edwards et al.^2^ noted that greater independence in activities of daily living, fewer neurological deficits, and better cognitive ability were the most common predictors of return to work. For promoting RTW, not only acute medical treatments but also comprehensive efforts including stroke and vocational rehabilitation, management, support by medical staff, and reception from patients with stroke are required.

Concerning RTW in patients with stroke, the health and employment support (HES) program was started in earnest at 12 Rosai (which means industrial accidents in Japanese) hospitals belonging to the Japan Organization of Occupational Health and Safety in 2015. The HES program provides these patients with dual support, such as acute medical treatments, stroke and vocational rehabilitation, management, and support by a dual support coordinator on the medical side; as well as management and support on the workplace side. In addition to stroke, other target diseases included in this program are cancer, diabetes, cardiac disease, and liver disease, to name a few.

Our medical center, one of the Rosai hospitals, participated in the HES program sponsored by the Japan Organization of Occupational Health and Safety in 2017. First, we conducted a single hospital study under the HES program^3^ on the RTW rate of 62 patients with acquired brain injury and revealed that the RTW rate was 81% and the RTW rates had no significant difference between the older group (age ≥ 65 years) and the working-age group (age < 65 years).

The purpose of the current study was to examine the rate of return to work for stroke patients desiring a return to work and receiving workplace interventions through the HES program in a large number of patients with stroke in plural Rosai hospitals, which were involved in the HES program sponsored by the Japan Organization of Occupational Health and Safety. The study also aimed to evaluate patient characteristics as predictors of outcomes and to assess whether age was a negative predictive factor for RTW.

## Patients and methods

We conducted a retrospective cohort study on RTW among inpatients with stroke based on aggregated results of implementation reports from April 2015 to March 2021, which were sent to the organization headquarters from 29 Rosai hospitals of the Japan Organization of Occupational Health and Safety on a quarterly basis. The implementation report contained anonymous information regarding all the patients during the covered period who participated in the HES program: name of hospital, disease, approximate age (such as sixties), gender, date and process of intervention, contents of intervention, problem list in this program, plans and results to the problems (including mental and physical disabilities), date for RTW, and others. A dual support coordinator from each Rosai hospital was asked to report the implementation status of each patient to the organization’s headquarters.

The study protocol was approved by the Institutional Review Board of Moji Medical Center (01-15). The review board waived the requirement for written informed consent because it had already approved the study protocol of the original HES program and treatments, and the contents of the implementation reports we used in this study were completely anonymized. This study was conducted in accordance with the principles of the Declaration of Helsinki.

According to the implementation reports, 561 patients with stroke admitted to one of 14 Rosai hospitals, who were employed at stroke onset and were willing to work again, were registered to participate in the HES program between April 2015 and March 2019. After excluding 78 patients due to other health problems, 23 for death, and 55 for unknown outcomes, the final number of participants was 483.

We collected the following information from the implementation reports: approximate age, gender, occupation (white-collar or blue-collar), stroke type (cerebral infarction, cerebral hemorrhage, subarachnoid hemorrhage), activities of daily living (ADL), neurological findings, and progress and outcome of RTW. Neurological findings were classified as right/left hemiplegia, neuropsychological deficits, combined disability (hemiplegia with neuropsychological deficits), and others. Neuropsychological deficits included ideomotor apraxia, constructional apraxia, unilateral spatial neglect, and aphasia. Other physical symptoms included ataxia, dysarthria, visual field impairment, and gait disturbance without hemiplegia.

All patients included in this program were working prior to experiencing stroke. The primary endpoint was RTW after stroke, which was defined as a work duration in active employment of ≥ 1 month. This definition applied to short-term jobs, changing to other work, and labor redeployment within the company.

The HES program was based on the guidelines for dual support for the treatment and work life of patients with stroke published by the Ministry of Health, Labor and Welfare. This program applied to patients who were employed at the time of stroke onset and expressed an expectation to return to work (RTW). The guidelines included medical treatments by neurologists; stroke rehabilitation by physiatrists, physical therapy, occupational therapy, and speech therapy; counseling, management, and support by medical social workers; and management and support in the workplace by occupational physicians, occupational health nurses, and the office staff in charge of RTW as needed. A specific feature of this program was the participation of dual-support coordinators who completed a 1-day course sponsored by the Japan Organization of Occupational Health and Safety.^4^ Most of the dual support coordinators were medical social workers, who were involved in exchanging information between the patient’s hospital and workplace, promoting cooperation, and adjusting the RTW plan. In the protocol, participants in this program received medical treatments and workplace interventions from dual-support coordinators during their admission. After being discharged either directly to their homes or through a rehabilitation hospital, participants were subsequently followed up by the coordinators until RTW. Participants who succeeded in RTW were followed up until the month of RTW, and the others were followed up for a maximum of 24 months. Reports detailing the progress towards RTW were submitted to the organization’s headquarters semiannually and the process was monitored.

Statistical analyses were performed using GraphPad Prism version 7 (GraphPad Software, La Jolla, CA, USA). Successful and unsuccessful RTW for each factor were examined using a χ^2^ test to determine the within-factor differences. The Kaplan–Meier method was used to calculate the curves of the RTW rates to examine differences in stroke types and neurological findings. A log-rank test was used to confirm between-group differences in the curves. Statistical significance was set at *P* < 0.05.

## Results

**Figure 1:**
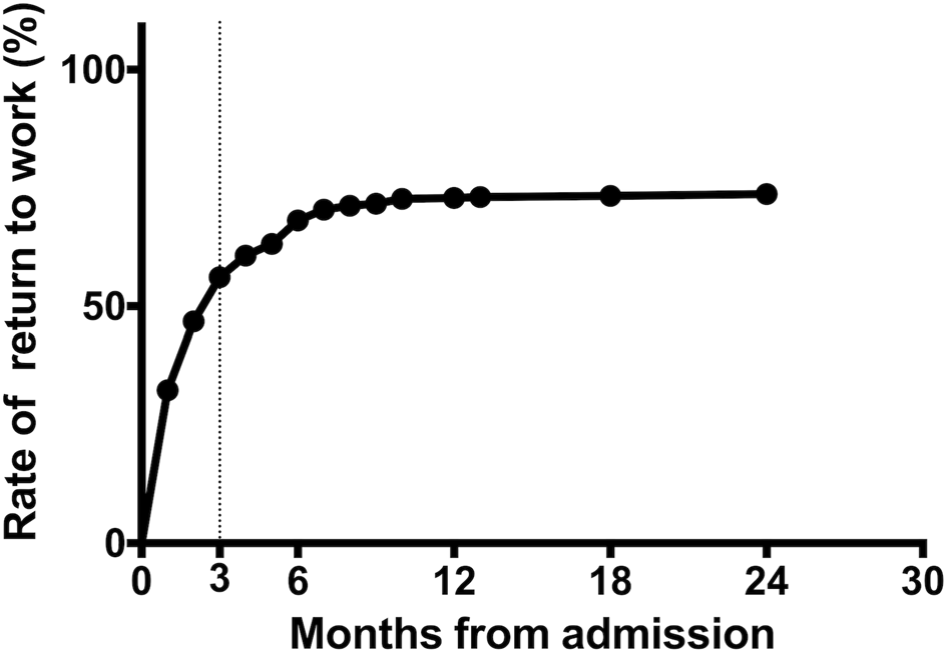
Return to work rate curve for all patients.

A total of 483 patients (377 men, 106 women; age 20–80 years, median age 50 years) underwent acute medical treatments and stroke rehabilitation during their hospital stay and participated in the HES program. Of these patients, 271 (56%) succeeded in RTW by the third month after stroke onset, 329 patients (68%) by the sixth month, and, finally, 355 (73%) by the 24th month (Fig. 1).

Table 1 shows that stroke types, neurological findings, and ADL were significant factors associated with RTW (χ^2^ test, *P* < 0.05), although age, gender, and occupation types (white-collar and blue-collar) were not significant factors (χ^2^ test, *P* > 0.05).

**Table 1:** Factors associated with return to work.

Factor	No.	Successful return to work	Unsuccessful return to work	*P* value
Age (years)				
< 60	300	221 (74%)	79 (26%)	0.914
≥ 60	183	134 (73%)	49 (27%)	
Gender				
Male	377	281 (75%)	96 (25%)	0.330
Female	106	74 (70%)	32 (30%)	
Occupation types				
White-collar	204	149 (73%)	55 (27%)	0.844
Blue-collar	279	206 (74%)	73 (26%)	
Stroke types				
Cerebral infarction	292	231 (79%)	61 (21%)	0.0003
Cerebral hemorrhage	141	86 (61%)	55 (39%)	
Subarachnoid hemorrhage	50	38 (76%)	12 (24%)	
Neurological findings				
Hemiplegia or/and neuropsychological deficits*	355	242 (68%)	113 (32%)	0.0001
Other symptoms^†^ or no deficits	128	113 (88%)	15 (12%)	
Activities of daily living				
Independence	413	344 (83%)	69 (17%)	0.0001
Dependence	70	11 (16%)	59 (84%)	

The χ^2^ test was used to determine differences in return to work by each factor.

*Ideomotor apraxia, constructional apraxia, unilateral special neglect, and aphasia.

^†^Physical symptoms other than hemiplegia such as ataxia, dysarthria, visual field impairment, and gait disturbance without hemiplegia.

**Figure 2:**
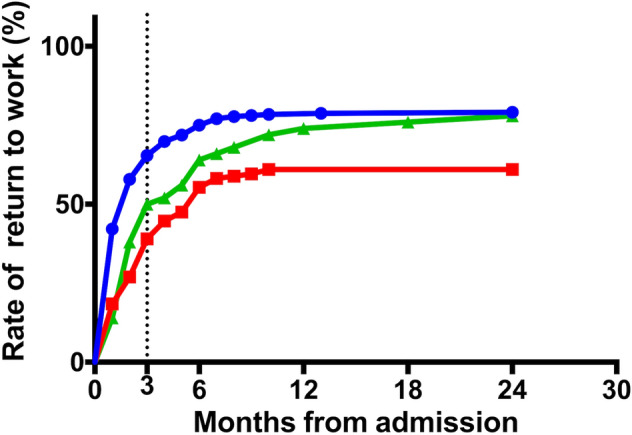
Return to work rate curves for the stroke types. Cerebral infarction has a significantly higher return to work rate curve than cerebral hemorrhage (Kaplan–Meier method, log-rank test, *P* < 0.01). Cerebral infarction: blue; cerebral hemorrhage: red; subarachnoid hemorrhage: green.

Figure 2 shows three curves of the RTW rate for different stroke types (cerebral infarction, cerebral hemorrhage, and subarachnoid hemorrhage) obtained using the Kaplan–Meier method. A significant difference in the RTW rate curves was found between cerebral infarction and hemorrhage (log-rank test, *P* < 0.01).

**Figure 3:**
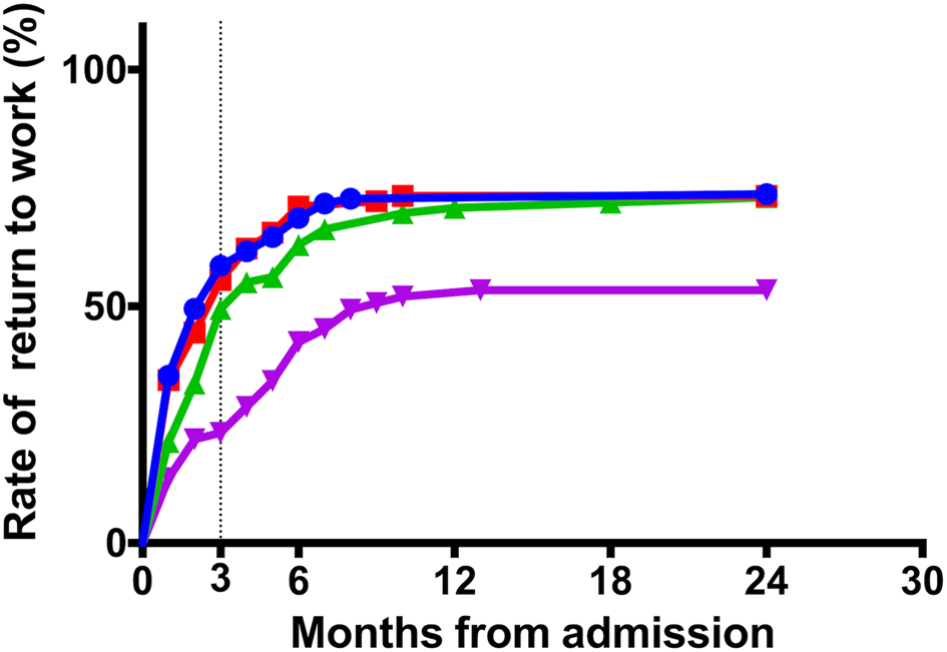
Return to work rate curves for the four neurological findings. Left hemiplegia: blue; right hemiplegia: red; neuropsychological deficits: green; hemiplegia with neuropsychological deficits: purple. There are significant among-group differences (log-rank test, *P* < 0.01).

Hemiplegia and neuropsychological deficits in the neurological findings in Table 1 were subclassified into four types, as shown in Table 2: left hemiplegia, right hemiplegia, neuropsychological deficits, and combined disability (hemiplegia with neuropsychological deficits). The type of neurological finding was a significant factor associated with RTW (χ^2^ test, *P* < 0.01). Figure 3 presents the profile of RTW rate curves of the four types of neurological findings by the Kaplan–Meier method with a significant difference (log-rank test, *P* < 0.01).

**Table 2:** Rates of return to work by neurological findings.

	No.	Successful return to work	Unsuccessful return to work	*P* value
Neurological findings				
Left hemiplegia	100	73 (73%)	27 (27%)	
Right hemiplegia	90	66 (73%)	24 (27%)	0.0005
Neuropsychological deficits	89	64 (72%)	25 (28%)	
Combined disability*	76	37 (49%)	39 (51%)	

*Hemiplegia with neuropsychological deficits.

The χ^2^ test was used to determine differences in return to work based on neurological findings.

## Discussion

This multicenter study on the HES program revealed that 73% of the 483 patients with stroke returned to the workplace, and age was not a significant factor for successful RTW. These results support the finding of our recent single-center study indicating that the RTW rate of 62 patients with acquired brain injury was 81%, and that older patients attained a successful RTW that was no less than that of working-age patients.^3^

The RTW rate in this study was considerably high, as compared with the findings of previous studies in our country: 58% of 244 patients after stroke between January 1986 and December 1990^5^ and 55% of 325 patients after stroke between February 2006 and July 2007.^6^ Saeki et al.^7^ found that the RTW rates were almost similar despite the progress in stroke treatment and rehabilitation, such as the use of tissue plasminogen activator, the introduction of convalescent rehabilitation wards, and public long-term care insurance. According to a recent systematic review by Edwards et al.^2^, RTW increases with time, with the median frequency increasing from 41% between 0 and 6 months, 53% at 1 year, 56% at 1.5 years, and 66% between 2 and 4 years post-stroke. In the field of occupational therapy, encompassing vocational rehabilitation and workplace intervention, Baldwin and Brusco reported work rates after completion of vocational rehabilitation ranging from 12 to 49% in a systematic review.^8^ Ntsiea et al.^9^ observed that workplace intervention involving workability assessments and workplace visits was effective in facilitating RTW for stroke survivors, and that the RTW rate at six months follow-up of the intervention group was 60% compared to that of the control group without workplace intervention, which was 20%. Toyota et al.^10^ documented the effectiveness of the HES program on RTW for stroke patients, noting an RTW rate of 67.7% in their Japanese article. These articles suggest that occupational therapy with a workplace intervention such as the HES program could yield high RTW rates. Information sharing between hospitals and workplaces was important for stroke survivors, as it led to the understanding of patients’ disabilities in workplaces, helping increase RTW rates. Nevertheless, the RTW rate of this study was high.

The reasons the RTW rate in this study was higher could be as follows:

1. All participants in this study expressed an expectation of returning to work. Westerlind et al.^11^ reported an RTW rate of 85% among 1695 participants of working age (18–58 years) who were working prior to stroke, and self-expectations of RTW were identified as a significant factor influencing the likelihood of RTW (odds ratio: 3.7). Conversely, in the systematic review conducted by Baldwin and Brusco, the pre-stroke vocational status of participants ranged from 48 to 100%, and self-expectations of RTW were not addressed.^8^ These factors might facilitate optimized rehabilitation after stroke and increase the RTW rate.
2. Cerebral infarction cases increased in number. Toyota,^12^ who analyzed 150,899 patients with stroke treated at 32 Rosai hospitals across Japan from 1984 to 2009, reported that the incidence of cerebral infarction increased annually from 54.6 to 66.2% until approximately 2003. Patients with cerebral infarction often show mild hemiplegia compared to patients with cerebral hemorrhage, and, therefore, the RTW rate in total may have become greater after 2003, showing an upward shift of the RTW rate curve yielded by the Kaplan–Meier method (Fig. 2).
3. Greater independence in ADL. The rate of independence in ADL in this study was 86%, which was high as compared with those in two previous studies: 59%^5^ and 24%.^7^ As Edwards et al.^2^ noted that greater independence in ADL was one of the most common predictors of RTW, the possibility of successful RTW would naturally become greater in patients who were independent in ADL.^1,6,13^
4. Mild disability of stroke. One of the most consistent predictors of RTW was stroke severity, and patients with mild to moderate stroke were more likely to RTW.^13^ However, because it is complicated to evaluate the severity of stroke including several functions in all body portions, a simple instrument for global disability evaluation, such as the modified Rankin Scale, is often used for the evaluation of outcomes or classification of severity of stroke. Lower scores on the modified Rankin Scale, indicating no to slight disability, were positively associated with an individual’s ability to RTW.^13^ Based on a reanalysis of the SUMO study,^14^ Toyota^12^ wrote that approximately 70% of 1,959 patients with stroke in their 30 s to 60 s recovered to have scores from 0 to 2 of the modified Rankin Scale at 3 months after onset. In this study, the RTW rate curve (Fig. 1) demonstrated that 56% of the patients attained RTW within 3 months post-stroke, indicating an early RTW.^6^ Under these circumstances, patients in our study who showed an early RTW may have had no to mild global disability and may have increased the RTW rate.
5. Positive effect of the HES program. The HES program implemented in this study may have influenced the patients with stroke toward positive RTW, although details remain to be elucidated. Saeki et al.^6^ performed a cohort study on the RTW at 21 Rosai hospitals between February 2006 and July 2007, and we also conducted a retrospective cohort study at 14 Rosai hospitals between April 2015 and March 2019 under the HES program. Although the RTW rate increased by 18% in the second study and independence in ADL also improved as mentioned above, we cannot presume that the HES program directly accelerated recovery of hemiplegia and functional disabilities. Considering that the Ministry of Health, Labor and Welfare and the Japan Organization of Occupational Health and Safety recommend the HES program for patients after stroke, patients, and their families, more patients after stroke with no or mild functional disabilities may have joined the support program.

Regarding participant severities, each rate of the modified Rankin scale (mRS) (0–5) could not be determined because the implementation report did not include the mRS. However, Matsutani et al. conducted an RTW program using almost the same method as that in this study, involving 61 patients, and reported that the RTW rate of patients with moderate stroke (mRS: 3, 4) was 80%. Therefore, this study was considered to include patients with moderate disabilities and not only mild stroke.^15^ Further research is necessary to reveal how the HES program improved the RTW rate.

In this study, age, gender, and occupation type were not significant factors for RTW after stroke. However, it has been reported that age less than 65 years^1^ and white-collar employment^1,5^ are positive factors for RTW, and being female^6^ is a negative factor for RTW.

According to the 2019 data from the Organization of Economic Co-operation and Development,^16^ the labor force participation rate of persons aged 65 years and above was increasing and reached 25.3% (rate of labor force aged ≥ 65 years within the same age group) in Japan. Given the recent changes in the aging Japanese population, there have been concomitant changes in employment among older adults and the ratio of men and women in the workplace.^17,18^ Umemura et al.^19^ reported that nonagenarians with stroke who presented with mild symptoms tended to have a good prognosis and returned to daily life just as younger patients. Therefore, age would not be a barrier to RTW alone if an older patient with normal cognitive functions had no or mild physical disability.

Regarding occupational types, Ashley et al.^13^ reported that individuals who worked in manual labor occupations were less likely to RTW, and Saeki et al.^5^ noted that being a white-collar worker was a significant positive predictor of RTW. Our previous single-hospital study on RTW showed that white-collar workers returned to their workplace more than blue-collar workers;^3^ however, no significant difference was found between both groups in this larger-scale study. Given the above-mentioned circumstances, age, gender, and occupational type may not be robust negative predictors of successful RTW.

The RTW curve of this study plateaued 12 months after stroke onset (Fig. 1). The RTW rate of participants with neuropsychological deficits increased mildly after 12 months (Fig. 3). Therefore, these curves suggest that support for participants' RTW should be continued for at least one year from stroke onset.

Hemiplegia with neuropsychological deficits (Tables 1, 2) and ADL (Table 1) were significant factors for RTW in this study, and the findings and ADL derived from the severity of stroke. Edwards et al.^2^ noted that greater independence in ADL, fewer neurological deficits, and better cognitive ability were the most common predictors of RTW, and we also consider these three as robust factors.

This study has several limitations. First, it was a retrospective cohort study based on implementation reports from 29 Rosai hospitals to determine the RTW rate. Because these reports were not medical records, we could not obtain details of neurological findings, scores of cognitive functions, or scores of physical disability evaluations. Second, the specific components of the HES program were not quantitatively measured, and the degree of adherence to the guidelines across participating hospitals was not examined. Third, all the participants in this study expressed an expectation to return to work, potentially introducing a selection bias for RTW outcomes, as this study could not include patients who had to abandon the prospect due to severe disabilities. Finally, we did not directly compare the HES program with ordinary stroke rehabilitation, and the effectiveness of the HES program remains to be investigated.

## Conclusion

Under the HES program, 73% of 483 patients after stroke attained RTW. Age, gender, and occupational types were not associated with successful RTW. Cerebral hemorrhage and hemiplegia with neuropsychological deficits were negative predictors of RTW, and independence in ADL was a positive predictor.

## Data Availability

The datasets analyzed in the current study are available from the corresponding author upon reasonable request.
